# Anatomy of the coronary sinus with regard to cardiac resynchronization therapy implantation

**DOI:** 10.1007/s00399-022-00863-x

**Published:** 2022-06-01

**Authors:** Rasmus Borgquist, Lingwei Wang

**Affiliations:** 1 Department of Clinical Sciences, Cardiology, Lund University, Lund, Sweden; 2 Department of Arrhythmias, Skane University Hospital, 221 85 Lund, Sweden

**Keywords:** Biventricular pacing, Left ventricular lead implantation, Thebesian valve, Vieussen’s valve, Vein of Marshall, Biventrikuläre Stimulation, Linksventrikuläre Elektrodenimplantation, Thebesische Klappe, Vieussen-Klappe, Marshall-Vene

## Abstract

Knowledge of the coronary sinus (CS) anatomy is crucial for implantation of cardiac resynchronization therapy (CRT). Obstacles to CS entry, such as the Eustachian ridge and Thebesian valve, as well as within the CS, such as Vieussen’s valve and the vein of Marshall, are important to understand and differentiate during implantation or to identify earlier by imaging. Anatomic knowledge is mandatory to select the most suitable side branch for lead implantation. Modern tools and techniques almost always enable other anatomic problems, such as tortuous, small, short, or overly straight side branches, to also be overcome.

## Abstract (de)

Kenntnisse der Koronarsinus(CS)-Anatomie sind von zentraler Bedeutung bei der Implantation von Systemen für die kardiale Resynchronisationstherapie (CRT). Hindernisse am CS-Ostium, z. B. eine Eustachi- oder Thebesische Klappe, und innerhalb des CS, z. B. eine Vieussen-Klappe oder Marshall-Vene, müssen während der Implantation erkannt und von anderen Hindernissen unterschieden oder zuvor durch Bildgebung identifiziert werden. Anatomisches Verständnis ist eine Voraussetzung, wenn der optimale CS-Seitenast für die Elektrodenimplantation ausgewählt werden soll. Moderne Materialien und Techniken erlauben es fast immer, auch anatomische Probleme wie kurvige, kleine, kurze oder zu gerade Seitenäste zu überwinden.

Cardiac resynchronization therapy (CRT) is an established treatment for heart failure in selected patients and has formed part of the European Guidelines on cardiac pacing in heart failure for almost 20 years [1, 2]. The first case reports of biventricular pacing appeared in the late 1990s and showed promising results [3–5]. However, early attempts were hampered by the lack of specific devices tailored for biventricular pacing and lack of suitable tools for cannulation of the coronary sinus (CS), which allowed for both easy cannulation and the introduction of a left ventricular (LV) lead. Nevertheless, technical development was fast, and dedicated CRT pacemakers (CRT-P) and defibrillators (CRT-D) were introduced and approved by regulatory authorities. This set the stage for landmark prospective randomized trials, such as the MUSTIC, Care-HF, and COMPANION trials, which were later followed by the MADIT-CRT and RAFT trials [1, 6–9].

There are several technical challenges to overcome in order to achieve reliable LV pacing via the epicardial CS branches. Success rates have improved over time, as experience and technical tools have developed. In the early Care-HF study, the implant success rate at first attempt was 89%, and successful implants included 12% of the leads placed in the great cardiac vein (i.e., on the epicardial surface of the interventricular septum, thereby not directly stimulating the LV free wall) [10]. The reason for failed implant was primarily failure to cannulate the CS or failure to achieve a stable lead position, but also included perforation or dissection of the CS.

The introduction of modern bipolar or quadripolar leads, including active fixation, has made it possible to reach a stable position even in CS branches that are relatively short and therefore do not accommodate an LV lead with traditional curved passive fixation. Recently published experience from a large volume tertiary care center with experienced implanters reported an initial success rate of 99%, and active fixation leads had a lower dislodgement rate at 6 months (0.74% vs. 4.69%, *p* = 0.036) [11].

Successful CRT implants depend on a number of factors, related to either the implanting physician (such as implanter experience and skill), the implant tools, or patient characteristics (anatomic variants). This review focuses on the anatomical aspects of the CS, as seen from a CRT implanter perspective.

## Normal anatomy

**Fig. 1:**
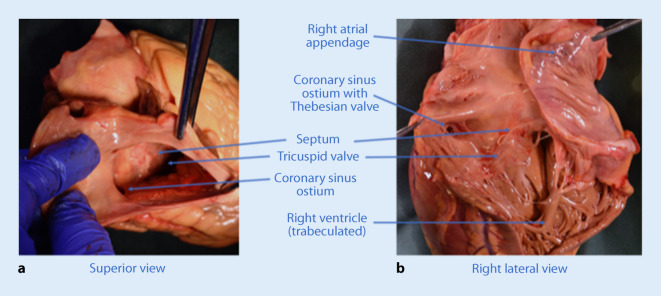
**a** The coronary sinus ostium and septal aspect of the right side of the heart seen from a superior view through the superior vena cava. Anteriorly is the tricuspid valve and the short distance between the valve and the coronary sinus can be appreciated. In **b** taken from the lateral side of the right ventricle, the relationship between the septal leaflet of the tricuspid valve and the coronary sinus ostium is more visible

A thorough anatomical knowledge is paramount in order to achieve a high success rate for CS implants. The CS ostium is normally located in the lower portion of the posteroseptal aspect of the right atrium (Fig. 1).

**Fig. 2:**
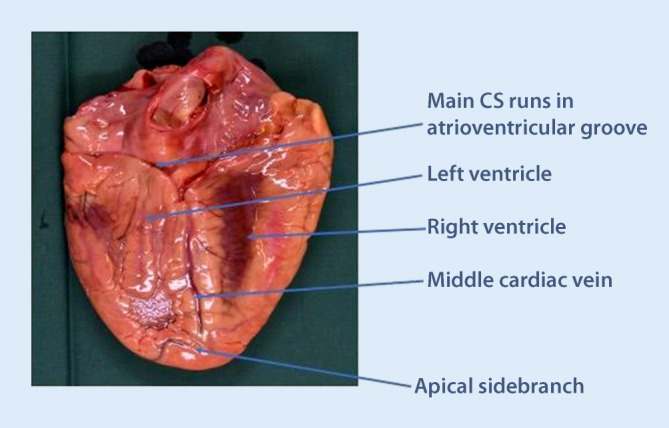
The outside of the infero-posterior aspect of the heart, showing the middle cardiac vein running in the interventricular groove, and the main coronary sinus (*CS*) running in the atrioventricular groove. The middle cardiac vein has a small apical side branch with an acute-angled take-off

The ostium lies between the septal aspect of the tricuspid valve, the fossa ovalis, and the inferior vena cava opening. On its way, it receives blood from several vein branches originating over the LV free wall, which are typically named after their location (i.e., posterolateral, lateral, anterolateral). The first branch (which can have a separate ostium) is usually the middle cardiac vein (MCV), which runs inferiorly in the interventricular groove to the apex of the heart (Fig. 2). The MCV may give off side branches that in theory can be used for lead placement, but usually these branches are very apical and thus not optimal for lead placement.

**Fig. 3:**
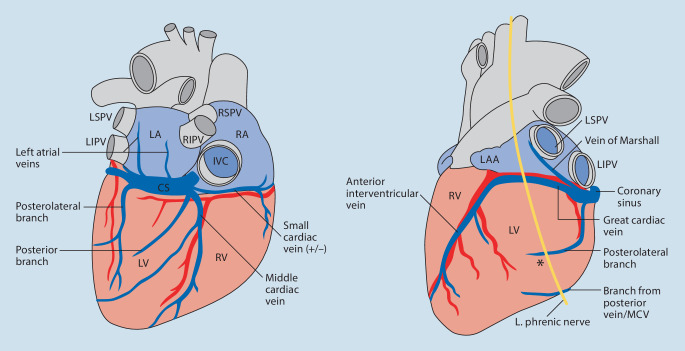
Schematic illustration of the coronary veins from a posterior view (**a**) and an anterior view (**b**). The *asterisk* represents a suitable lateral veinbranch where a lead can be placed. *LSPV* left superior pulmonary vein, *LIPV* left inferior pulmonary vein, *LA* left atrium, *RSPV* right superior pulmonary vein, *RIPV* right inferior pulmonary vein, *IVC* inferior vena cava, *LV* left ventricle, *CS* coronary sinus, *LAA* left atrial appendage, *RV* right ventricle

The main CS then runs posteriorly in the atrioventricular (AV) groove, and later becomes the great cardiac vein (GCV) at the level of Vieussen’s valve. It follows the AV groove to the anterior side of the heart and then runs on the superior aspect of the interventricular groove towards the apex, in parallel with the left anterior descending coronary artery (Fig. 3; [12]). The number of anatomically suitable vein branches traversing the LV between the MCV and the GCV varies, but on average there are between two and three potential vein branches that can be used [13].

**Fig. 4:**
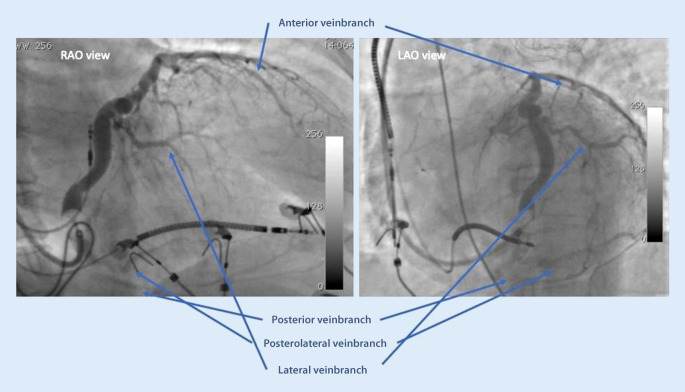
Venograms from the right anterior oblique (*RAO*) and the left anterior oblique (*LAO*) views

The nomenclature of the vein branches can be somewhat confusing, especially since cardiologists have traditionally used the names posterior, posterolateral, lateral, anterolateral, and anterior for branches in the respective territories [14], whereas in echocardiography, cardiac computed tomography (CT) and cardiac magnetic resonance imaging (MRI), the traditional 17-segment model is used [15]. It is important to recognize that in order to determine the location of a pacing electrode tip in a particular cardiac segment, it is necessary to view two perpendicular X‑ray or fluoroscopy angles—the left anterior oblique (short axis view) and the right anterior oblique (long axis view) (Fig. 4). In the anteroposterior view alone, it is impossible to determine the location of a specific cardiac segment.

## Anatomical challenges for the implanter

The anatomical challenges can be divided into the following parts:

### 1. CS access

i. Right atrial enlargement
ii. Thebesian valve
iii. Anatomical variants
  - CS stenosis
  - Persistent left superior vena cava with or without CS atresia and unroofed CS

### 2. Vein-branch access

i. Vieussen’s valve and the vein of Marshall
ii. Separate ostium of posterior or posterolateral branch
iii. Tortuous and small caliber veins
iv. Phrenic nerve stimulation

### 1. CS access

**Fig. 5:**
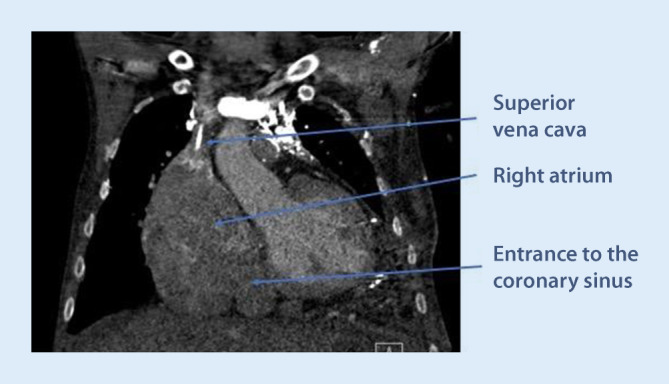
Preoperative cardiac computed tomography showing extreme enlargement of the right atrium, with the coronary sinus (CS) ostium in the usual position in the lower medial corner of the atrium. In such a case, a standard CS catheter often has insufficient support to enter the CS, and larger or stiffer sheaths need to be used
ii. The CS ostium may be partly, sometimes completely, covered by the Thebesian valve, which is a thin fibrous structure that may be fenestrated in some cases. Together with the extension of the Eustachian valve, the extension of the Thebesian valve forms a small ridge (Eustachian ridge) running in the right atrium, posterior to and in parallel with the tricuspid annulus. Together with the septal leaflet of the tricuspid valve and the extension of the inferior vena cava valve (the tendon of Todaro), the Eustachian ridge delineates the triangle of Koch, where the AV node is typically located. The ridge is derived from the valve of the embryonic sinus venosus (which is the origin of the valves of the CS and inferior vena cava). The Eustachian ridge may effectively prevent soft cannulation catheters from entering the CS, by deflecting them in a more posterior direction when trying to enter from the atrial side. This problem is best avoided by first entering the right ventricle with the catheter, and then retracting it during counter-clockwise torque. By using this technique, the ridge can actually be of help by guiding the catheter towards the CS ostium once it flips out from the right ventricle through the tricuspid valve.
iii. The Thebesian valve can vary in configuration and texture. While it usually does not hamper CS cannulation, it may in some cases be very prominent and impossible to pass using a soft sheath. A prominent Thebesian valve can be seen on cardiac CT or suspected during implant if no or very little contrast enters the CS (Fig. 6). At times, a guidewire can pass through a fenestration, making it easy to advance the wire but impossible to advance the sheath after the wire, since the sheath becomes entangled in the valve around the fenestration. This problem can be overcome by using a steerable solid electrophysiology catheter either on the side or inside the soft sheath. The EP catheter can be manipulated to deflect the valve to allow for the soft sheath to advance into the CS.

i. Before entering the CS ostium, the catheter or sheath needs to be advanced through the right atrium. With a severely enlarged right atrium, there is less or no support for the catheter if resistance is encountered at the CS ostium (Fig. 5). Therefore, the cardiac dimensions need to be reviewed prior to implant, and special tools may be required in the case of right atrial enlargement. Congenital disease such as Ebstein’s anomaly may also alter the location of the CS ostium. In patients with congenital malformations that could involve the atrium or venous system, a pre-operative cardiac CT is strongly recommended. An example is shown in Fig. 5, where there is extreme enlargement of the right atrium, but CT images show that there is still a patent entrance to the coronary sinus system in the usual location (Fig. 5).

**Fig. 6:**
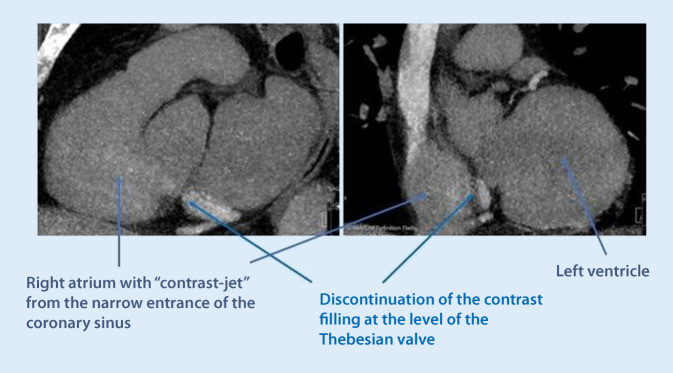
Cardiac computed tomography showing the right atrium and ostium of the coronary sinus. There is contrast passage from the coronary sinus (CS) to the atrium, but the prominent Thebesian valve appears as a discontinuation of the CS right at the level of the ostium
iv. Anatomical variants may affect the success rate of CS cannulation and lead placement. Narrowing of the CS ostium is not an uncommon finding and, in some cases, makes it impossible to pass with a sheath. This often occurs secondary to previous lead placements or other device placement in the CS, but can also occur without prior interventions. The problem can be overcome by either progressive balloon dilatation with a percutaneous coronary intervention (PCI) technique, or by using a stable steerable sheath that is positioned immediately outside the ostium, providing enough support for a sub-select catheter to enter. One alternative is to use a large 9‑F inner-diameter sheath outside the ostium and place a superstiff 0.035 in wire deep into the CS to keep the sheath stabilized and in place. Assistance in the CS cannulation via simultaneous femoral access with a diagnostic EP catheter may also increase the success rate of CS cannulation and lead placement in these difficult cases (Fig. 7).

**Fig. 7:**
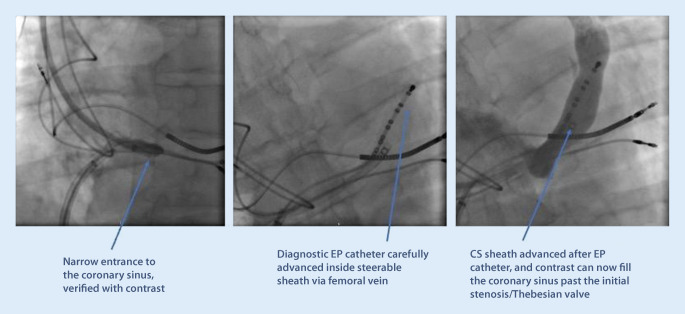
An example of difficult cannulation of the coronary sinus (CS) due to proximal stenosis and prominent Thebesian valve. By using a diagnostic electrophysiology catheter to pass through the initial part of the CS, keeping it “open,” a standard CS sheath can then be advanced in parallel to the EP catheter, gaining access for lead placement
  Using the steerable catheter with real-time electrogram guidance during CRT implantation makes location of and access to the CS more rapid and successful, thus improving the success rate of CRT implantation and saving implantation and fluoroscopy times [16].
  A persistent left superior vena cava (PLSVC) is the most common thoracic venous anomaly and may also be a component of complex cardiac pathologies. The prevalence of PLSVC ranges from 0.2 to 0.5% in the general population, but in patients with congenital heart disease between 1.3 and 11%. It may be accompanied by CS ostial atresia, in which case the PLSVC becomes the major retrograde drainage pathway for the coronary veins and there is secondary enlargement of the CS. In some cases, there is a direct connection between the CS and the left atrium (defined as an unroofed CS). In such a case, it is important to appreciate that this represents a potential right-to-left shunt and therefore permanent anticoagulation is indicated if a CS lead is placed. It is technically challenging to place an LV lead via a PLSVC, since the angle of take-off is usually steep and the enlarged main CS does not provide sufficient support. It is therefore recommended to use the right vena cava access for CS cannulation, unless there is CS atresia.

### 2. CS branch access

**Fig. 8:**
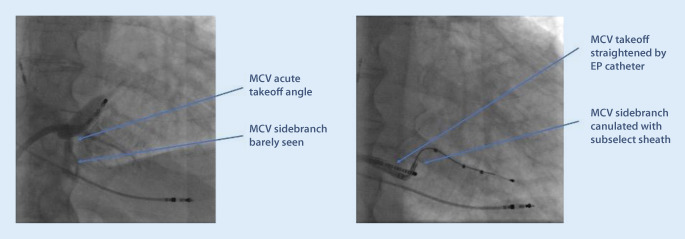
Difficult cannulation of an early side branch to the middle cardiac vein (*MCV*). The MCV take-off is highly angulated from the main coronary sinus, and there is another 90° turn to enter the side branch. An electrophysiology catheter and a subselect sheath are used for gaining access to the target vein (in this case, no other options were available due to no capture or phrenic nerve stimulation)

**Fig. 9:**
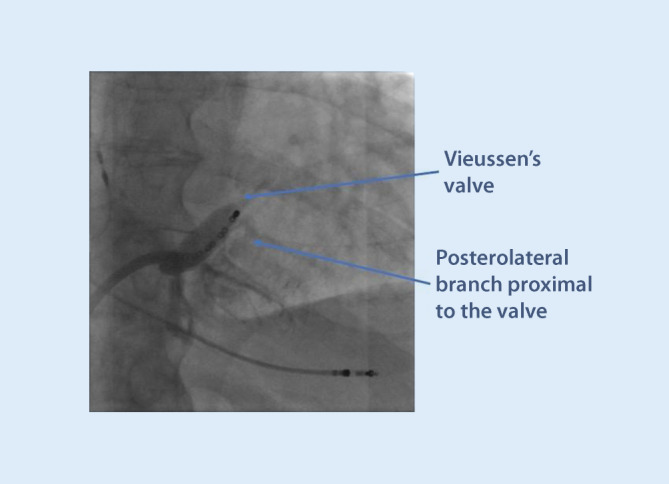
Forceful contrast injection ends abruptly and fails to fill the more distal parts of the coronary sinus (CS) due to a prominent Vieussen’s valve, which obstructs retrograde passage through the main CS lumen

**Fig. 10:**
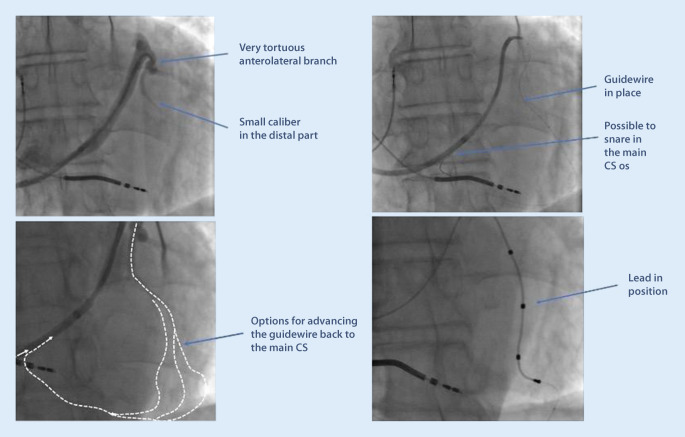
A case with very tortuous anatomy of the only available (anterolateral) side branch. Small collaterals connected to the proximal main coronary (*CS*) trunk, and by using these for snaring, a left ventricular lead was successfully placed in a lateral position

i. Difficult ostium entrance. A common ostium for the MCV and the main CS can confuse the implanter as to where the sheath should be advanced. While the lateral branches from the main CS are usually preferred for LV lead placement, a side branch to the MCV can certainly be used as a second option in some cases. It is not uncommon that there is a tortuous take-off of the MCV from the common ostium, and cannulation can be challenging (Fig. 8).
  By using slight clockwise torque from the ostium, it is often possible to pass a soft subselector or guidewire into the branch. If the side branch is short or without angle, lead stability may be an issue, and an active fixation lead is preferred.

ii. Vieussen’s valve. The valve is situated where the main CS transforms into the GCV, which then continues to the superior part of the interventricular septum. It is composed of between one and three leaflets and is present in 80–90% of people. Normally, the leaflets are soft, non-occlusive, and easy to pass, but the valve can in rare cases be a challenging obstacle to pass with the sheaths and the electrode [17]. A sign of a prominent Vieussen’s valve is if contrast does not pass deeper into to CS when injected from a catheter from within the main CS trunk (Fig. 9). Immediately before the valve, there may be a patent vein of Marshall (the remnant distal part of the fetal left anterior cardinal vein), taking off in a superior direction and draining the left atrium. Vieussen’s valve may be associated with a curve of the CS, rendering its passage more difficult. Usually, it does not present a challenge for the CRT implanter, but there is the risk that the vein of Marshall is mistaken for the main CS trunk, and a balloon is inflated for an occlusive venogram. The vein of Marshall will not accommodate an inflated balloon, will burst and a tamponade may result if balloon inflation is attempted.
  Using 4‑F catheters with a soft angulated tip in combination with hydrophilic wires, it is usually possible to get past the valve and then advance a subselect sheath further into a lateral branch. Often it is not necessary to pass the valve if there is a suitable posterolateral branch with take-off proximal to the valve.
iii. Tortuous and small caliber vein branches are a common challenge for the implanter, and an array of tools may be needed to overcome this problem. Sometimes, the take-off is at an atypical angle, making it hard to engage the vein at all with a wire. Sometimes, the take-off is easy, but there is a tortuous course of the vein, making it impossible to advance sheaths or an electrode over the wire. Step one in this situation should always be to perform a venogram and make sure that there are no better options available in other branches. The use of a subselector sheath facilitates engagement of acute-angled vein branches, but is not always helpful if the tortuosity is more distal. Working with larger diameter subselectors allows for multiple guidewires to stabilize the sheath before advancing it. Other techniques involve snaring the guidewire through a collateral in the main CS (Fig. 10) or using the balloon anchoring technique. If the vein branch is too short to allow for proper anchoring of a passive curved fixation LV lead, active lead fixation may be used in the main CS, just letting the distal part protrude into the short vein branch.
iv. The left phrenic nerve runs adjacent to the pericardium, and the exact course is unpredictable. Phrenic stimulation can occur in any of the lateral segments, and sometimes prevents delivering of LV pacing altogether. However, with the introduction of quadripolar leads, the problem has diminished considerably. By trying different vectors of stimulation, it is usually possible to find a configuration with capture that does not give rise to phrenic stimulation. In addition, most of the patients with CRT are not pacemaker-dependent, and low margins of safety for the LV lead can therefore be accepted if the threshold is stable.

## Practical conclusion

This review presents the normal anatomy of the CS and its tributary vein branches from an electrophysiologist’s perspective. The most common anatomic variants and technical pitfalls for the implanting physician have been highlighted and concise guidance presented along with tips and tricks for overcoming these challenges. EP is a rapidly evolving field, and as more tools and implanting techniques become available, it is essential for every implanting physician to stay updated with the latest developments. A fundamental understanding of the cardiac anatomy is a prerequisite for becoming a successful CRT implanter.

## Declarations

For this article no studies with human participants or animals were performed by any of the authors. All studies mentioned were in accordance with the ethical standards indicated in each case.
